# HIV-1 with HBV-associated Q151M substitution in RT becomes highly susceptible to entecavir: structural insights into HBV-RT inhibition by entecavir

**DOI:** 10.1038/s41598-018-19602-9

**Published:** 2018-01-26

**Authors:** Yoshiaki Yasutake, Shin-ichiro Hattori, Hironori Hayashi, Kouki Matsuda, Noriko Tamura, Satoru Kohgo, Kenji Maeda, Hiroaki Mitsuya

**Affiliations:** 1 Bioproduction Research Institute, National Institute of Advanced Industrial Science and Technology (AIST), Sapporo, 062-8517 Japan; 2 National Center for Global Health and Medicine Research Institute, Tokyo, 162-8655 Japan; 3 Faculty of Pharmaceutical Sciences, Sojo University, Kumamoto, 860-0082 Japan; 4 Experimental Retrovirology Section, HIV and AIDS Malignancy Branch, National Cancer Institute, National Institutes of Health, Bethesda, MD 20892 USA; 5 Department of Clinical Science, Kumamoto University Hospital, Kumamoto, 860-8556 Japan

## Abstract

Hepatitis B virus (HBV) reverse transcriptase (RT) is essential for viral replication and is an important drug target. Nonetheless, the notorious insolubility of HBV RT has hindered experimental structural studies and structure-based drug design. Here, we demonstrate that a Q151M substitution alone at the nucleotide-binding site (N-site) of human immunodeficiency virus type-1 (HIV-1) RT renders HIV-1 highly sensitive to entecavir (ETV), a potent nucleoside analogue RT inhibitor (NRTI) against HBV. The results suggest that Met151 forms a transient hydrophobic interaction with the cyclopentyl methylene of ETV, a characteristic hydrophobic moiety of ETV. We thus solved the crystal structures of HIV-1 RT^Q151M^:DNA complex with bound dGTP or ETV-triphosphate (ETV-TP). The structures revealed that ETV-TP is accommodated at the N-site slightly apart from the ribose ring of the 3′-end nucleotide, compared to the position of bound dGTP and previously reported NRTI/dNTP. In addition, the protruding methylene group of bound ETV-TP directly pushes the side-chain of Met184 backward. Met184 is a key residue that confers ETV resistance upon substitution with smaller Ile/Val. These results provide novel insights into NRTI binding to the N-site and further provide important clues for the development of novel anti-HBV/HIV-1 RT inhibitors to overcome critical drug resistance.

## Introduction

Hepatitis B virus (HBV), a small DNA virus affecting approximately 400 million people worldwide, causes acute and chronic hepatitis, resulting in approximately 1 million deaths annually^1^. HBV is replicated in the nucleocapsid core particle by reverse transcription from an RNA intermediate, a process catalyzed by virally encoded polymerase (Pol)^2–4^. HBV Pol is a unique enzyme with a molecular weight of approximately 90 kDa and contains 4 functionally distinct domains: terminal protein (TP), spacer, polymerase, and ribonuclease H (RH), which accomplishes RNA-dependent DNA polymerization via a tyrosine residue derived from the TP domain as a protein primer^5,6^. High-resolution structural information on HBV Pol has long been sought to facilitate anti-HBV drug development, as well as to understand the molecular mechanism of self-primed reverse transcription initiation. However, obtaining sufficient soluble and catalytically active recombinant HBV Pol for crystallographic study remains extremely challenging^7^.

**Figure 1:**
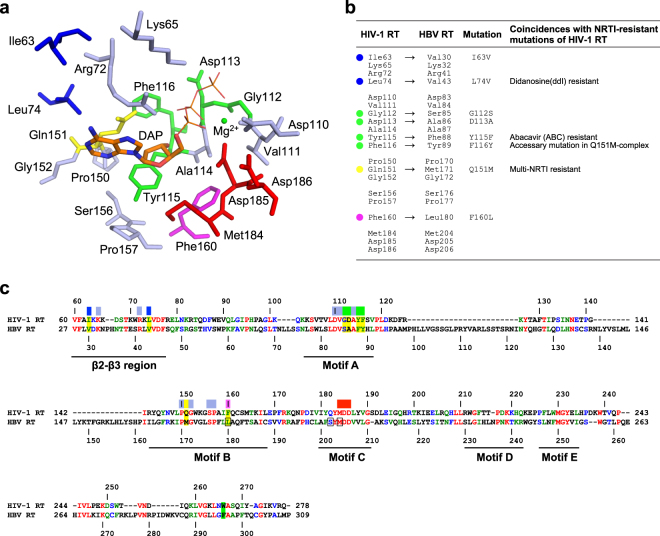
Design of HIV-1 RT mutants mimicking the N-site of HBV RT in this study. (**a**) The residues lying within 7 Å of the deoxyribonucleoside moiety of the bound NRTI/dNTP determined by previously reported ternary complexes of HIV-1 RT. This diagram was drawn using the structure of HIV-1 RT^WT^ in complex with DNA and dATP (PDB code, 5TXL)^11^. The deoxyadenosine moiety of the bound dATP (DAP) is shown in stick model. The unconserved 8 residues selected for mutational analysis are colored in blue (Ile63 and Leu74), green (Gly112, Asp113, Tyr115 and Phe116), yellow (Gln151) and magenta (Phe160). (**b**) The 20 selected residues in HIV-1 RT and the corresponding residues in HBV RT. The 8 residues selected for mutational analysis are marked with the same color scheme as in (**a**). Coincidences with NRTI resistance of HIV-1 RT are also indicated. (**c**) Amino acid sequence alignment between HIV-1 RT and HBV RT. HIV-1 and HBV RT domains were extracted and used for the alignment. The identical, strongly similar, and weakly similar residues are colored in red, green, and blue, respectively. The residues selected for mutational analysis in this work are highlighted in yellow. The conserved motifs (β2-β3 and motifs A, B, C, D, and E) are indicated. The color bars above the sequence indicate the 20 selected residues with the same color scheme as in (**a**). The residues involving ETV resistance in HBV RT are boxed. Residue stacking at the ribose ring of primer DNA at the N + 3 position shown in Fig. 6 is highlighted in green.

Currently, all approved chemotherapeutics for anti-HBV treatment comprise nucleoside analogue reverse transcriptase (RT) inhibitors (NRTIs)^8^. NRTIs are tri-phosphorylated intracellularly into nucleotides, acting as a chain terminator(s) by tightly binding to the deoxynucleotide-triphosphate (dNTP)-binding site (N-site) of HBV RT. Although the amino-acid sequence similarity between HBV and human immunodeficiency virus type-1 (HIV-1) RT is very low (approximately 8%), several moderately conserved (around 35% sequence identity) motifs in the restricted regions that form the N-site have been identified (Fig. 1). Particularly, it should be noted that a glutamine residue (Gln151) of HIV-1 RT located at the entrance of the N-site is, without exception, substituted to bulky, hydrophobic methionine in HBV RT. Additionally, several residues near the N-site are similar but not identical between HIV-1 and HBV RTs (Fig. 1 and Supplementary Fig. S1). Conceptually, HBV and HIV-1 RTs share common metal-dependent dNTP-binding and catalytic mechanism of nucleotide addition to the 3′-end of the primer DNA, whereas amino acid differences around the N-site probably result in different NRTI sensitivities. Notably, HIV-1 RT Q151M is a critical mutation that confers multi-NRTI resistance, accompanied by A62V, V75I, F77L, and F116Y mutations (Q151M-complex)^9,10^. Recently, the structures of HIV-1 RT^Q151M^ and RT^Q151M-complex^ have been reported and suggest that Q151M leads to conformational perturbation of the HIV-1 RT N-site^11^.

**Figure 2:**
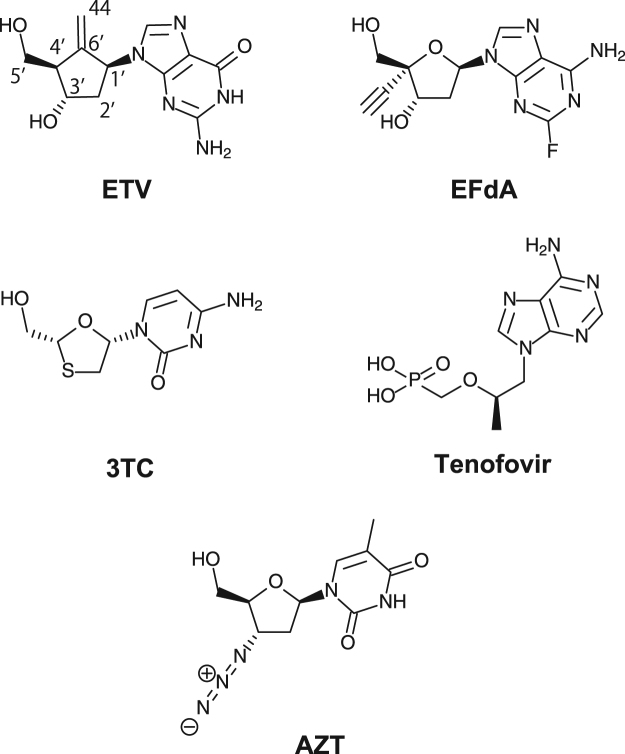
Chemical structures of NRTIs used in the antiviral assay. ETV, entecavir; 3TC, lamivudine; AZT, zidovudine. TDF is a prodrug, and only the acylic nucleoside phosphonate moiety of TDF (i.e., tenofovir) is shown.

Entecavir (ETV), a carbocyclic 2′-deoxyguanosine analogue, is currently the most potent anti-HBV drug (Fig. 2)^12,13^. ETV is reportedly also active against HIV-1, albeit at very low levels compared to those against HBV. Most NRTIs lack the 3′-OH group and thus act as obligate chain terminators, whereas ETV contains a 3′-OH group that allows the addition of several nucleotides following ETV-monophosphate (ETV-MP) incorporation before chain termination. The phenomenon of RT inhibition by ETV occurs in both HIV-1 and HBV^14,15^ and is referred to as “delayed chain termination” which enables evasion of incorporated NRTI phosphorolytic excision, a major NRTI-resistance mechanism of HIV-1 RT^16^. Recently, multiple ETV-resistant HBV RT mutations (i.e., L180M, S202G and M204I/V) have been reported; therefore, novel HBV RT inhibitors are necessary to develop combination therapies against chronic HBV infections to hamper ETV-resistant HBV mutant selection^17,18^. It is important to elucidate the interatomic interactions between ETV-triphosphate (ETV-TP) and HBV RT by X-ray crystallography to understand the structural mechanism of HBV RT inhibition and the reported ETV resistance in detail, which may also be useful for novel HBV RT inhibitor design. Until now, docking simulation studies with homology models based on the HIV-1 RT structure have been conducted to explain the putative interactions between ETV-TP and HBV RT^18,19^.

In this study, we showed that the Q151M mutation of HIV-1 RT confers high ETV and EFdA sensitivity upon HIV-1. The HIV-1 RT^Q151M^ further enabled crystal structure analysis of HIV-1 RT in complex with ETV-TP. Based on the structures of RT^Q151M^ together with the results of the antiviral assay, possible mechanisms of ETV-TP action on HIV-1/HBV RT and of the reported ETV resistance are discussed.

## Results and Discussion

### Viral replication and antiviral assay

Based on the previously reported ternary complex structures of HIV-1 RT, a total of 20 amino acid residues lying within 7 Å of the deoxyribonucleoside moiety of the bound NRTI/dNTP were extracted (Fig. 1). Among the selected residues, 8 were not conserved in HBV RT; 6 were located at the N-site of HIV-1 RT, whereas the other two were located at the β2-β3 region of the finger domain and stabilize the base moiety of the template DNA for base-pairing with incoming NRTI. These 8 unconserved residues were finally selected for the HBV RT-mimicking mutational strategy (Fig. 1 and Supplementary Fig. S1). Of these 8 mutation candidates, we reasoned that substitution of the hydrophilic Gln151 for the bulky and hydrophobic methionine (Q151M) would be the most significant, while the remaining 7 candidates were categorized as mutations with similar side-chain properties. It should also be noted that Q151M is widely known as a multi-NRTI-resistant mutation in HIV-1 RT, as described later. We created 5 HBV RT-mimicking HIV-1 RT mutants in this study: RT^Q151M^, RT^Q151M/F160L^, RT^Q151M/G112S/D113A^, RT^Q151M/Y115F/F116Y^, and RT^Q151M/I63V/L74V^ (Table 1). Wild-type HIV-1 (HIV-1^WT^) and the 5 HIV-1 variants carrying RT N-site mutations (e.g., HIV-1^Q151M^) were employed and their replication examined. Their replication kinetics suggested that although HIV-1^Q151M/F160L^ completely failed to replicate when cultured up to 9 days, the other 4 variants successfully propagated (Supplementary Fig. S2). HIV-1^Q151M^, HIV-1^Q151M/Y115F/F116Y^, and HIV-1^Q151M/I63V/L74V^ propagated slower than did HIV-1^WT^, but HIV-1^Q151M/Y115F/F116Y^ reached similar or slightly greater supernatant p24 levels on day 9 than did HIV-1^WT^. These HIV-1 variants were considered able to propagate, but their replication was somewhat slower than that of HIV-1^WT^. Notably, the magnitude of enzymatic activity of RT^Q151M^ was almost identical to that of RT^WT^ (Supplementary Table S1), and further investigation will be needed to explain the slight difference between HIV-1 RT activity and the propagation profile of HIV-1 with the Q151M mutation. In contrast, the propagation of HIV-1^Q151M/G112S/D113A^ was relatively slow, with a day 9 viral level approximately 40% that of HIV-1^WT^. Taken together, the results implied that the F160L mutation was exceptionally harmful to viral replication.

**Table 1:** HBV RT-mimicking HIV-1 RT mutants created in this study.

Mutants	Motif B		Motif A				β2-β3		Viral Replication
	Q151M	F160L	G112S	D113A	Y115F	F116Y	I63V	L74V	
RT^Q151M^	°								++
RT^Q151M/F160L^	°	°							−
RT^Q151M/G112S/D113A^	°		°	°					+
RT^Q151M/Y115F/F116Y^	°				°	°			++
RT^Q151M/I63V/L74V^	°						°	°	++

Viral replication represents the viral replicability obtained from the results of viral replication kinetics as shown in Supplementary Fig. S2.

We chose 3 well-replicable variants (HIV-1^Q151M^, HIV-1^Q151M/Y115F/F116Y^, and HIV-1^Q151M/I63V/L74V^) for the antiviral assay, and investigated whether these HBV RT-mimicking mutations could change sensitivities against the typical anti-HBV/HIV-1 NRTIs (Fig. 2): entecavir (ETV, anti-HBV NRTI), 4′-ethynyl-2-fluoro-2′-deoxyadenosine (EFdA, anti-HIV), lamivudine (3TC, anti-HIV/HBV), tenofovir disoproxil fumarate (TDF, anti-HIV/HBV), and azidothymidine (AZT, anti-HIV). As summarized in Table 2, all NRTIs showed antiviral activity against HIV-1^WT^; EFdA was most potent (IC_50_: 0.4 nM), followed by AZT (7 nM) and TDF (62 nM). In previous studies, ETV was reportedly inactive against HIV-1, but has subsequently shown minor activity against HIV-1^20,21^. We also confirmed that ETV was active against HIV-1^WT^, albeit with the lowest activity among the tested compounds (IC_50_: 1.1 μM).

**Table 2:** Results of the anti-viral assay using typical NRTIs.

Agents	IC_50_ (μM)			
	HIV-1^WT^	HIV-1^Q151M^	HIV-1^Q151M/Y115F/F116Y^	HIV-1^Q151M/I63V/L74V^
ETV	1.1 ± 0.37	0.042 ± 0.015 (26.2)	0.013 ± 0.004 (86.4)	0.390 ± 0.211 (2.8)
EFdA	0.0004 ± 0.0001	0.00003 ± 0.00001 (13.3)	0.00003 ± 0.00002 (13.3)	0.00008 ± 0.00002 (5.0)
3TC	0.393 ± 0.021	0.085 ± 0.028 (4.6)	0.069 ± 0.037 (5.7)	0.629 ± 0.083 (0.63)
TDF	0.062 ± 0.01	0.058 ± 0.003 (1.1)	0.056 ± 0.032 (1.1)	0.024 ± 0.019 (2.6)
AZT	0.007 ± 0.006	0.034 ± 0.018 (0.21)	0.205 ± 0.121 (0.034)	0.032 ± 0.013 (0.22)

Parentheses refer to the relative drug sensitivity values calculated as IC_50_^WT^/IC_50_^Mutants^.

Next, we examined the antiviral activity of these NRTIs against HIV-1^Q151M^, HIV-1^Q151M/Y115F/F116Y^, and HIV-1^Q151M/I63V/L74V^. As Shirasaka *et al*. and Harada *et al*. reported, HIV-1 RT Q151M, known as a multi-NRTI-resistant mutation, usually occurs in combination with accessory mutations, including A62V, V75I, F77L, and F116Y (Q151M-complex). The Q151M-complex causes high-level resistance to AZT and certain NRTIs with the dideoxy configuration, such as ddC and ddI^10,22^. Notably, Q151M is also an important AZT-resistant mutation to HIV-2 RT^23,24^. We confirmed that HIV-1^Q151M^ also caused moderate-level resistance to AZT (~5-fold); the combination of Q151M and F116Y decreased AZT sensitivity further, as expected (IC_50_: 0.2 μM) (Table 2). Notably, mutations including Q151M and some accessory mutations are also reportedly cause intermediate-level resistance to 3TC and TDF^25^; however, such resistance was not caused by any variants tested in this study (Table 2). Critically, EFdA activity against HIV-1^Q151M^ and HIV-1^Q151M/Y115F/F116Y^ increased (IC_50_: 30 pM for both variants) when compared to that against HIV-1^WT^ (IC_50_: 0.4 nM). Furthermore, the anti-HIV-1 activity of ETV drastically improved in the presence of Q151M and additional mutations Y115F and F116Y; the IC_50_ numbers of ETV against HIV-1^Q151M^ and HIV-1^Q151M/Y115F/F116Y^ decreased to 42 nM and 13 nM, respectively (26-fold and 85-fold less than that of HIV-1^WT^). This suggested that the Q151M mutation was strongly associated with ETV-TP binding to the N-site of HIV-1 RT, with additional Y115F/F116Y mutations further enhancing the ETV sensitivity of HIV-1^Q151M^. Considering the results of the viral replication and antiviral assays, we reasoned that HIV-1 RT^Q151M^ would be a suitable candidate for crystallographic study to elucidate the HIV-1 RT N-site structure in the ETV-TP binding state.

### Structure determination of RT^Q151M^ binary and ternary complex

Recombinant HIV-1 RT^WT^ and RT^Q151M^ were produced by *Escherichia coli* and purified by Ni-affinity and ion-exchanging chromatography (see Methods). The RT assay for the purified sample by enzyme-linked immune-sorbent analysis (ELISA) demonstrated that RT^Q151M^ and RT^WT^ showed similar activity levels (Supplementary Table S1). Quantitation by polymerase chain reaction (PCR) using viral supernatant of 293 T cells showed that RT^Q151M^ activity was approximately 77% that of RT^WT^ (Supplementary Table S1). These results indicated that the Q151M mutation does not significantly affect the enzymatic activity of HIV-1 RT.

**Figure 3:**
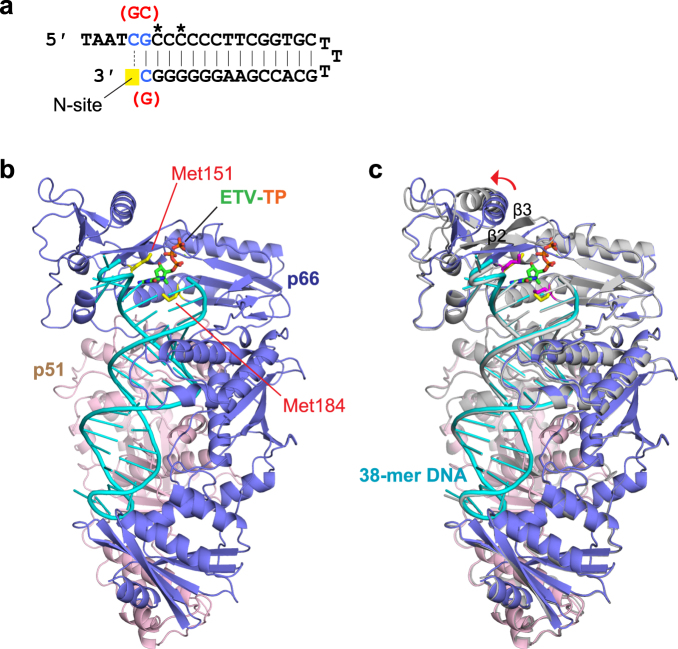
RT^Q151M^:DNA complex preparation and structure determination. (**a**) The 38-mer DNA aptamer used in this study. The nucleotides with 2′-O-methyl modification are marked with asterisks. Nucleotide substitutions are colored in blue, and the positions adjusted for the HIV-1 RT N-site are highlighted in yellow. The original nucleotides reported by Miller *et al*.^27^ are also indicated in red. (**b**) Ribbon representation showing the overall structures of the RT^Q151M^:DNA:ETV-TP ternary complex, and (**c**) the superimposition of the RT^Q151M^:DNA binary and RT^Q151M^:DNA:ETV-TP ternary complexes. The p66, p51, and DNA of the ternary complex are colored in blue, pink, and cyan, respectively. All models of the binary complex are colored in gray. The side-chains of Met151 and Met184 of the ternary complex are shown as stick models and colored in yellow. The side-chains of Met151 and Met184 of the binary complex are shown in magenta. The bound ETV-TP is also shown as a stick model. The structural difference at the finger domain including the β2-β3 region is indicated by a red arrow.

For crystallization of the RT^Q151M^:DNA binary complex, the previously developed 38-mer DNA aptamer was applied, albeit with 3 base substitutions to trap guanosine analogue ETV-TP at the N-site (Fig. 3a)^26,27^. After mixing RT^Q151M^ and the DNA aptamer, gel-filtration chromatography was applied to separate unbound DNA and the RT^Q151M^:DNA complex. Native polyacrylamide gel electrophoresis (PAGE) analysis showed that the peak fractions contained no DNA-free RT^Q151M^, indicating that the RT^Q151M^:DNA was stable and well equilibrated (Supplementary Fig. S3). The structure of the RT^Q151M^:DNA binary complex was determined by molecular replacement with the previously reported RT^WT^:DNA binary complex (PDB code, 5D3G)^27^ as a search model and refined to 2.6 Å resolution (Fig. 3b and c). There are two RT^Q151M^ heterodimers (p66/p51) in the asymmetric unit of the rhombohedral *R*3 lattice. The final refined model contains amino acid residues 3–553 for chains A/C (p66 subunit), 4–427 for chains B/D (p51 subunit) except 214–230 owing to poor electron density, and two DNA aptamers (nucleotides from −1 to 33 for chain E and from −4 to 33 for chain F). In the previously reported RT^WT^:DNA structure, the side-chain of Gln151 interacted with the adenine base of the dA0 via ordered solvent molecules, stabilizing base stacking with the cytosine base of dC1^27^. In contrast, the present structure showed that the hydrophobic side-chain of Met151 keeps nearby residues, the base moiety of dC0, and solvent away, and that the cytosine base of dC0 is deviated by approximately 35° relative to the guanine base of dG1. Consequently, the present structure of the RT^Q151M^:DNA binary complex exhibits a more open conformation than that of the previously reported RT^WT^:DNA structure (Supplementary Fig. S4). These results indicated that the bulky, hydrophobic Met151 side-chain increases the flexibility of the N-site and forms a distinct conformational state in the absence of dNTP/NRTI.

**Figure 4:**
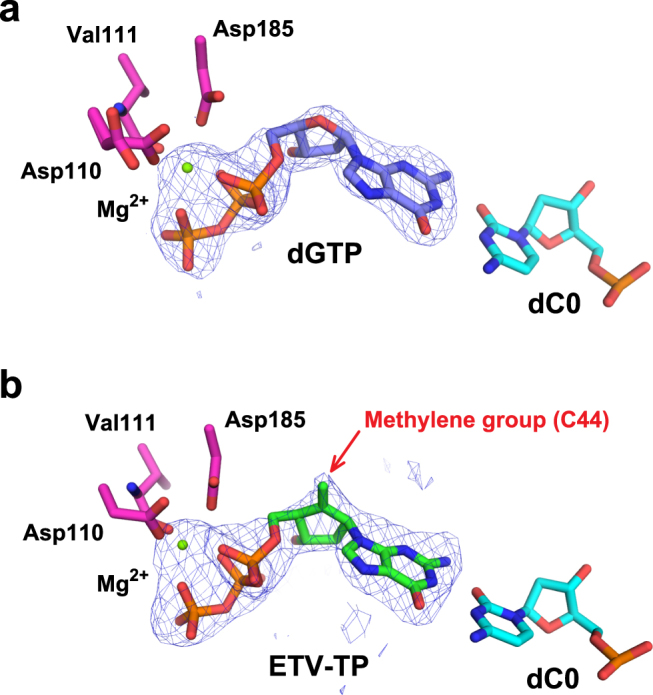
Simulated annealing *F*_o_ − *F*_c_ omit map for the bound Mg^2+^:dGTP (**a**) and Mg^2+^:ETV-TP (**b**). The metal-coordinating residues (Asp110, Asp185, and Val111) and the template nucleotide base-pairing with dGTP/ETV-TP (dC0) are shown as stick models. The map is contoured at the 2.6σ level. The exocyclic methylene in ETV-TP is indicated by a red arrow.

**Figure 5:**
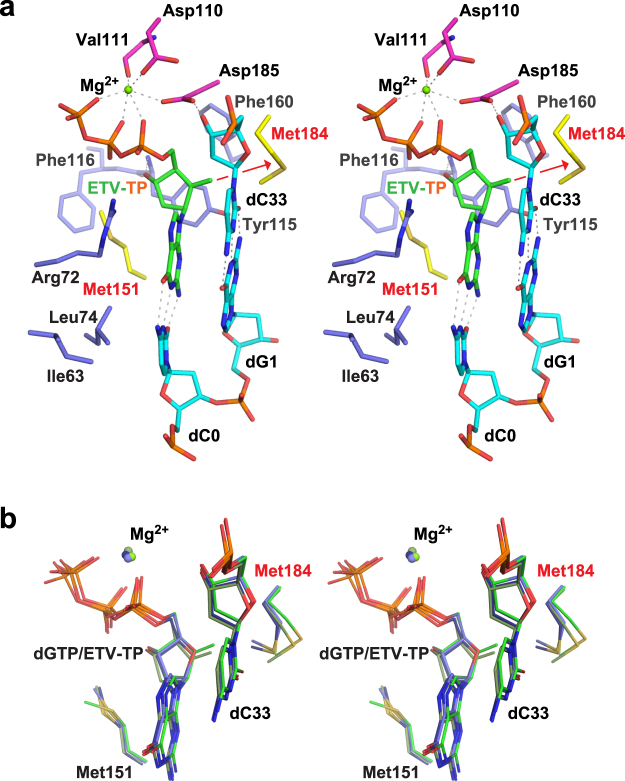
Stereo view of the N-site structure of the ternary complex. (**a**) RT^Q151M^:DNA:ETV-TP. Carbon atoms are colored as follows: important Met residues (Met151 and Met184), yellow; metal-chelating Asp110, Asp185, and Val111, magenta; other nearby residues interacting with ETV-TP, light blue; DNA, cyan; ETV-TP, green. Specific interatomic contacts (hydrogen bonds) are indicated as dotted lines. The methylene group of ETV-TP pushes the side-chain of Met184 backward as indicated by the red arrow. (**b**) N-site superimposition of ETV-TP and dGTP ternary complexes. Carbon atoms of ETV-TP complex chain A, ETV-TP complex chain C; dGTP complex chain A, and dGTP complex chain C are colored in green, dark green, blue, and dark blue, respectively.

RT^Q151M^:DNA:dGTP and RT^Q151M^:DNA:ETV-TP ternary complex crystals were prepared by soaking the crystals of the binary complex in the crystallization mother liquor supplemented with dGTP/ETV-TP. The structures of the RT^Q151M^:DNA:dGTP and RT^Q151M^:DNA:ETV-TP ternary complexes were determined at 2.38 Å and 2.45 Å resolution, respectively. The obvious electron density was observed for the bound dGTP/ETV-TP and Mg^2+^ (Fig. 4). The two heterodimers in the asymmetric unit are well superimposed on one another with main-chain root-mean-square deviation (RMSD) below 1.0 Å. In contrast, apparent local conformational changes were observed in the β2-β3 region of the finger domain between the binary and ternary complexes with maximum atomic displacement of approximately 8.5 Å (Fig. 3c). The Arg72 and Lys65 side-chains, derived from the β2-β3 region, interact with the bound ETV-TP to stabilize the ternary complex during catalytic reactions (Fig. 5). In RT^WT^ structures, a hydrogen bond is formed between the side-chain of Gln151 and Arg72, whereas the thioether group of Met151 in the RT^Q151M^ structures occupies roughly the same position as Gln151 but keeps the distances of van der Waals contacts with nearby Arg72, Phe115, and C2′ of dGTP/ETV-TP (3.6−4.0 Å). The same observations have also been made in the recently reported structures of HIV-1 RT^Q151M^ and RT^Q151M-complex^ ternary complexes^11^. The structures reported in this work have been determined in the rhombohedral lattice system for the first time, whereas the structures of RT^Q151M^:DNA:dGTP/ETV-TP basically exhibit a typical closed conformation of the HIV-1 RT ternary complex, and thus superimpose well on previously reported structures of the dNTP/NRTI ternary complex with main-chain RMSD of ~1.2 Å^11,28,29^. These results suggest that the lattice forces and the inter-molecular crystal contacts do not affect the overall structural conformation of HIV-1 RT ternary complex.

### Structural description of the RT^Q151M^ N-site occupied by ETV-TP

The overall conformation of the present ternary complex and the position and orientation of bound dGTP/ETV-TP are similar to those in previously reported HIV-1 RT:dNTP/NRTI complexes, whereas slight but significant structural differences at the N-site occupied by ETV-TP were also detected. First, the ribose-analogous cyclopentane ring of the bound ETV-TP lies slightly apart from the 3′-end nucleotide of the primer DNA. Second, relocation of the side-chain of Met184 occurs by pressing with the protruded exocyclic methylene group of the ETV-TP (Fig. 5, Supplementary Figs S5 and S6). The side-chains of Gln91 and Gln161 also moved in response to the relocation of the side-chain of Met184, retaining appropriate interatomic distances. We selected and listed two interatomic distances in Supplementary Table S2 as representative of the observed differences in the present structure of the ETV-TP ternary complex. In previous docking simulation studies, the exocyclic methylene of ETV-TP was predicted to be fitted with the small hydrophobic pocket created by Ala87, Phe88, Pro177, Leu180, and Met204 of HBV RT^19^. The corresponding hydrophobic pocket is observed in the present structure of the ETV-TP complex formed by Ala114, Tyr115, Pro157, Phe160, and Met184 of HIV-1 RT. However, the methylene did not exploit the pocket, but directly pushed the Met184 side chain (Fig. 5, Supplementary Figs S5 and S6). It is thus likely that the side-chain thioether of the Met184 plays a crucial role in ETV-TP acceptance through hydrophobic interaction with its methylene group, a characteristic moiety of ETV. Notably, Met184 of HIV-1 RT corresponds to Met204 of HBV RT, with M204I/V being a critical amino-acid substitution in acquiring ETV resistance. HIV-1 RT M184V was also detected from HIV-HBV co-infected patients receiving ETV monotherapy, and *in vitro* experiments further demonstrated that M184V of HIV-1 RT confers resistance to ETV^30^. The substitution of the bulky thioether group with a smaller isopropyl group could diminish contacts with methylene, leading to decreased binding affinity of ETV-TP to the N-site. Based on these results, we propose that modification of the ETV methylene moiety may represent a strategic candidate to develop new anti-viral agents for overcoming ETV resistance.

### Multi-step mechanism of RT inhibition by ETV-TP

**Figure 6:**
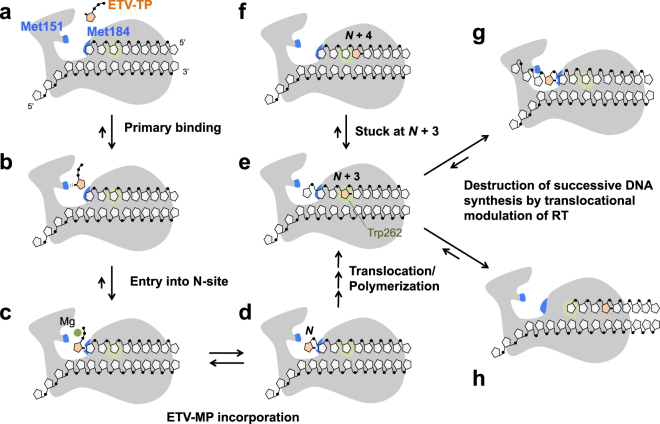
Proposed model for the mechanism of HIV-1/HBV RT inhibition by ETV-TP. Two Met residues of HIV-1 RT (Met151 and Met184) are labeled and colored in blue (**a**). ETV-TP is transiently trapped at Met151 (**b**), then enters into the N-site for tight binding, accepted by relocation of the Met184 side-chain, which is the state ready for ETV-MP incorporation (**c**). RT catalyzes the ETV-MP addition to the primer DNA 3′-end (**d**), and successive DNA synthesis continues. When ETV-MP is located at the N + 3 position, strong pausing of DNA synthesis occurs probably owing to DNA strand distortion that leads to 3′-end misalignment at the N-site (**e** and **f**). Trp262 of HIV-1 RT is located at the N + 3 position for stacking interactions with the ribose ring. The corresponding residue is substituted with Phe in HBV RT (Phe296). Successive DNA synthesis is finally stopped by translocational modulation of RT, leading to sliding over several nucleotides (**g** and **h**). The locations of the slipped DNA strand in (**g**) and (**h**) are drawn based on the results of RNase H cleavage analysis reported by Tchesnokov *et al*.^16^.

Although single Q151M substitution leads to a drastic increase in ETV sensitivity, the current structure of the ternary complex revealed no direct interactions between the Met151 side-chain and the exocyclic methylene of ETV-TP. It is likely that Gln151 does not impede the binding of ETV-TP to the N-site. These structural aspects as well as the results of antiviral assays strongly suggest that Q151M contributes not to the tight binding of ETV-TP to the N-site as observed in the crystal structure analysis, but to the process of ETV-TP entry into the N-site. Gln/Met151 is located at the entrance of the N-site and likely acts as a lid (Supplementary Fig. S4). Notably, Q151M also intensified sensitivity against EFdA by approximately 13-fold. EFdA has a characteristic ethynyl group at the C4′ position. The previously reported structure of the HIV-1 RT:DNA:EFdA-TP ternary complex revealed that the 4′-ethynyl is bound at a hydrophobic pocket created by the Ala114, Tyr115, Phe160, and Met184 side-chains^28^, yet does not directly interact with Gln151. ETV and EFdA share an increased hydrophobicity conferred by a methylene and ethynyl group, respectively. Therefore, we presumed that Met151 provides the primary binding site for ETV-TP/EFdA-TP through transient hydrophobic interactions, prior to the stable ternary complex formation observed in the crystal structures. The primary binding at Met151 might also stimulate local conformational change of the finger domain, thereby decreasing the energy barrier for entry of ETV-TP into the N-site. Collectively, we propose a three-step mechanism of ETV-TP action on HIV-1 RT: (i) primary binding to HIV-1 RT at Met151 via transient hydrophobic interactions; (ii) tight binding to N-site accepted by relocated Met184 side-chain, allowing incorporation of ETV-MP into primer DNA; and (iii) delayed chain termination at the N + 3 position as reported previously (Fig. 6)^16^. There might be a similar multi-step mechanism of ETV-TP action on HBV RT.

In recent structural studies of the HIV-1 RT^Q151M^ and RT^Q151M-complex^, a multi-NRTI resistance mechanism was proposed: the Q151M mutation increases the perturbation of the N-site by removal of the hydrogen bond between Gln151 and Arg72, leading to decreased incorporation of 3′-exo-forming dideoxy NRTIs^11^. We further propose the additional possibility that Gln/Met151 plays a role as a selector in response to the hydrophobicity of incoming NRTI. For example, Q151M confers resistance against AZT by 5-fold^31^. AZT has a highly polarized azide group at the C3′ position, which might not conform to the primary binding site provided by hydrophobic Met151, thus hindering the entry of AZT to the N-site and consequently decreasing binding affinity. Because the amino acid residues forming the N-site are identical between HIV-1 and HIV-2 RT, similar mechanisms, i.e., N-site perturbation and increased hydrophobicity caused by the Q151M mutation, likely underlie the AZT resistance of HIV-2 RT^Q151M^. Crystal structure analysis of HIV-2 RT^Q151M^:DNA:NRTI would be needed to discuss the NRTI-resistance mechanism of HIV-2 RT more precisely.

### Insights into the NRTI sensitivity and N-site structure of HBV RT

The structural information on HIV-1 RT^Q151M^ together with the results of the antiviral assays enable us to further discuss the individual role of each amino acid residue at or near the N-site in affecting NRTI sensitivity and resistance. The antiviral assay revealed that the Y115F and F116Y mutations increased the ETV sensitivity of HIV-1^Q151M^ by 3-fold. A recent structural study of HIV-1 RT^Q151M-complex^ suggested that the F116Y mutation improves polymerization fitness, which might be caused by N-site stabilization through hydrogen bond formation between the side-chain hydroxyl of Tyr116 and the main-chain O of Lys73^11^. The same stabilization effect is expected when ETV-TP is bound to the N-site of HIV-1 RT^Q151M/Y115F/F116Y^, thus leading to the increase of ETV sensitivity. In addition, Phe115 might be more suitable for stacking interaction with the cyclopentyl ring of ETV-TP than is Tyr115, which would also increases ETV-TP binding affinity. In contrast to Y115F/F116Y, the I63V/L74V mutations markedly decreased ETV sensitivity. These two residues are located near the cytosine base of dC0, and their side chains play a role in stabilizing base-pair formation with the guanine base of ETV-TP. I63V/L74V thus weaken the stabilization of bound ETV-TP at the N-site, which might drastically decrease ETV-MP incorporation into primer DNA. The results also suggested that local structures, including Ile63 and Leu74 (β2-β3 region), might differ considerably between HBV and HIV-1 RTs.

Definitive structural differences between HIV-1 RT^Q151M^ and HBV RT likely exist, because HIV-1 RT^Q151M^ exhibits high sensitivity to both ETV and EFdA. EFdA is not potent against HBV RT^32^. We speculate that the hydrophobic pocket of HIV-1 RT accommodating the unique 4′-ethynyl group of EFdA may be distorted or destroyed by F160L and neighboring G112S/D113A mutations, leading to the preclusion of EFdA-TP binding to the N-site of HBV RT. Unfortunately, HIV-1 could not be propagated upon introduction of the F160L mutation. Phe160 is located at the depth of the hydrophobic pocket adapted by EFdA 4′-ethynyl, and forms the hydrophobic core of the thumb domain with the side-chains of Met164 and Phe214, which are not conserved in HBV RT. Therefore, the local structure around Phe160 is likely an important factor yielding NRTI sensitivity differences between HIV-1 and HBV RT.

## Conclusion and perspectives

We generated 5 HIV-1 RT mutants to mimic the HBV RT N-site, constructing recombinant HIV-1 containing those HIV-1 RT mutants for viral replication and anti-viral assays, and found that the Q151M mutation of HIV-1 RT alone is critical for conferring high ETV sensitivity on HIV-1. The structure of ETV-TP is identical to that of dGTP, except that ETV-TP has a cyclopentyl methylene instead of a ribose ring oxygen. Therefore, the results of the antiviral assays suggest that the side chain of Met151 forms a transient hydrophobic interaction with the cyclopentyl methylene of ETV-TP; this interaction is apparently required for smooth ETV-TP entry into the N-site. We also determined the crystal structure of HIV-1 RT^Q151M^ in complex with DNA, DNA:dGTP, and DNA:ETV-TP, which revealed that ETV-TP is bound at the N-site, directly pushing the Met184 side chain. The corresponding Met204 substitution by the smaller Val/Ile, known as a major ETV-resistance mutation in HBV RT, could decrease the binding affinity of ETV-TP to the N-site. Among the total of 8 amino-acid substitutions introduced in this study (Table 1), Q151M, Y115F, F116Y, and L74V have been reported as mutations associated with NRTI resistance in HIV-1 RT (Fig. 1b). Therefore, exhaustive structural studies of these NRTI-resistant HIV-1 RT mutants in complex with various NRTIs would be helpful for further understanding of the relationship and differences in NRTI sensitivities between HIV-1 and HBV RT.

## Methods

### Viral replication kinetics and antiviral assays

The genes encoding HIV-1 RT p66 and p51 used in this study originated from the HIV-1 clone NL4–3 (GenBank: M19921.2). All site-specific mutations were introduced by inverse PCR^33^ using pCDF_p66 as a template DNA^34^. HIV-1_NL4-3_-based infectious clones with RT mutations were constructed using the In-Fusion HD Cloning Kit (Clontech). The In-Fusion enzyme fuses the PCR-generated DNA sequence (RT region with mutations) and a linearized vector (HIV-1_NL4-3_-based plasmid) by recognizing a 15 bp overlap at their ends. Viral population replication kinetics were measured as previously described^35^. In brief, each virus (wild-type or carrying RT mutations) was harvested by the transfection of HIV-1 plasmids to 293 T cells. MT-4 cells (10^4^, in a 96-well plate) were exposed to 200 TCID_50_ of each HIV-1 and cultured without antiretroviral agents for 9 days. No fresh MT-4 cells were added during the culture period. The p24 values in supernatants were determined on days 0, 3, 6 and 9. Antiviral assays (p24 assay) using wild-type HIV-1 (HIV-1^WT^) and replicable HIV-1 variants (HIV-1^Q151M^, HIV-1^Q151M/Y115F/F116Y^, and HIV-1^Q151M/I63V/L74V^) were also conducted as previously described^35^. In brief, MT-4 cells were exposed to a virus at 100 50% tissue culture infectious doses (TCID_50_s). After exposure, the cell suspension (5 × 10^3^ cells in 100 µL) was plated into each well of a 96-well culture plate containing various concentrations of drugs. After incubation for 7 days, the amounts of p24 antigen in supernatants were determined using a Lumipulse G1200 immunoassay system (Fujirebio) as previously described^36^ with minor modifications and the drug concentrations suppressing p24 Gag protein production by 50% (50% inhibitory concentration [IC_50_]) were determined by comparison with p24 production levels in drug-free control cultures. All assays were performed in duplicate.

### Synthesis of ETV-TP

ETV-TP was synthesized according to the scheme shown in Supplementary Fig. S7. First, hydroxyl groups of ETV **1** were protected as triethylsilyl ether. After protection of the *N*^2^-amino group with an isobutyryl group, the triethylsilyl group was removed to give compound **2**. Second, 5′- and 3′-hydroxyl groups were protected with 4,4′-dimethoxytrityl and isobutyryl groups, respectively. Deprotection of the 4,4′-dimethoxytrityl group gave a compound **3**. Third, phosphorylation of the 5′-hydroxyl group of compound **3** by the phosphoramidite method followed by deprotection gave ETV 5′-monophosphate **4** (ETV-MP). Finally, ETV-MP was converted to the corresponding 5′-triphosphate **6** (ETV-TP) by the phosphoroimidazolidate method, as previously described^37^.

### Protein expression and purification

HIV-1 RT expression vectors (pET28_His_6_-p51 and pCDF_p66) were prepared as previously described^34^. To increase the stability and crystallizability of the RT:DNA complex, two Cys residues were substituted with Ser (C162S and C280S) for both p51 and p66, the 4 C-terminal residues were truncated from p66, and the human rhinovirus 3 C protease cleavage sequence was inserted between His_6_ and the p51 gene fragment^38^. Recombinant HIV-1 RT^WT^ and RT^Q151M^ were overexpressed by *E. coli* strain BL21-CodonPlus (DE3)-RIL and purified as described^34^ with slight modification as follows. The crude extract was applied to a Ni-affinity column (Sigma-Aldrich) pre-equilibrated with buffer A (50 mM Na phosphate pH 8.0, 600 mM NaCl, 2 mM MgCl_2_, and 10% glycerol). The column was washed with buffer A followed by buffer B (same composition as buffer A but with pH 6.0); bound samples were eluted with a linear gradient of 0–400 mM imidazole in buffer B. The collected eluent was dialyzed against buffer C (50 mM Tris-HCl pH 8.0, 2 mM MgCl_2_, 1 mM dithiothreitol [DTT], and 10% glycerol) and loaded into a DEAE Sepharose Fast Flow column (GE Healthcare). The pass-through was collected, diluted with a 3-fold volume of buffer D (same composition as for buffer C but with pH 7.5), and concentrated 4-fold by Amicon Ultra centrifugal filters with 50 kDa cut-off (Millipore). The samples were further applied to a CM Sepharose CL-6B column (GE Healthcare), and the bound RT was eluted with a linear gradient of 0–400 mM NaCl in buffer D. The fractions containing equal molar of p66 and p51 were collected, dialyzed against a buffer consisting of 10 mM Tris-HCl pH 8.0 and 50 mM NaCl, and concentrated to 14 mg/mL using 50 kDa cut-off Amicon Ultra centrifugal filters.

### HIV-1 RT enzyme assays

The RT activity of HIV-1 RT^WT^ and RT^Q151M^ was determined using ELISA and PCR methods. ELISA was conducted using the Reverse Transcriptase Assay kit (Roche) according to the manufacturer’s instruction. Purified His-tagged enzymes produced by *E*. *coli* were used for the ELISA assay. For the PCR-based RT assay, cell-free viral supernatants from 293 T cells were collected 3 days after transfection with an HIV-1-coding plasmid, and a SYBR Green-based real-time PCR-enhanced RT (SG-PERT) assay was performed as previously described with slight modifications^39^. In brief, viral lysate was prepared by incubating the mixture containing cell-free viral supernatant and virus lysis buffer (0.5% Triton X-100, 1 mM MgCl_2_, 25 mM Tris-HCl and 1 mM DTT final concentrations) for 10 min at room temperature. RT reaction mixture (10 µL) containing the viral lysate, MuLV RT buffer (1×) (New England Biolabs), dNTPs (1 mM), RNase inhibitor (4 U) (TaKaRa Bio), MS2 reverse primer (500 nM), and 1 µL pre-heated (65 °C, 5 min) MS2 RNA (Roche) was prepared, and the following reactions were performed: 60 min RT reaction at 37 °C, 5 min RT inactivation at 95 °C. SYBR Green-based real-time PCR using the following MS2 primers (MS2 fwd: 5′-TCC TGC TCA ACT TCC TGT CGA G-3′, MS2 rev: 5′-CAC AGG TCA AAC CTC CTA GGA ATG-3′). RT activity standard curve values were determined by running a dilution series of commercial recombinant HIV-1 RT (Bio Academia) in parallel.

### RT^Q151M^:DNA complex formation

The previously established 38-mer hairpin DNA aptamer^27^ was employed for the structural study of the RT^Q151M^:DNA:dGTP/ETV-TP ternary complex with 3 base substitutions to accommodate dGTP and ETV-TP at the N-site (Fig. 3a). The 38-mer DNA was purchased from Hokkaido System Science, Co., Ltd. The DNA dissolved in TE buffer (10 mM Tris-HCl pH 8.0 and 1 mM EDTA) was heated at 80 °C for 10 min, then cooled slowly to 25 °C. The DNA and purified RT^Q151M^ were mixed and incubated overnight at 4 °C. The mixture was injected onto a HiLoad 16/600 Superdex 200 pg gel-filtration column (GE Healthcare) using a buffer consisting of 10 mM Tris-HCl pH 8.0, and 50 mM NaCl. Native PAGE with a Tris-borate buffer was performed to examine fraction contents and purity. The gel was initially stained by fluorescent dye (ATTO) for detection of DNA, followed by staining with Coomassie Brilliant Blue (CBB) for detection of proteins (Supplementary Fig. S3). The peak fractions were collected and concentrated to approximately 15 mg/mL for crystallization.

### Crystallization

All crystallization experiments were conducted by the vapor-diffusion technique at both 4 °C and 20 °C. Initial crystallization screenings using commercially available sparse matrix kits were performed by the sitting-drop vapor-diffusion method in 96-well plates. Drops consisted of equal volumes (100 nL) of sample and reservoir solution and were equilibrated against 70 μL of reservoir solution. Optimization of the initial hit condition was performed using hanging-drop vapor-diffusion with the 24-well plates. The hanging-drops were set up by mixing sample solution (1.0 μL) with reservoir solution (1.0 μL), and equilibrated against 500 μL reservoir solution. Crystals suitable for structure analysis were obtained at 20 °C using a reservoir solution consisting of 20 mM Bis-tris-HCl pH 6.0, 60 mM di-ammonium hydrogen citrate, 20 mM MgCl_2_, 1.5–3% PEG 6000, 2–4% sucrose, and 4–8% glycerol. The crystals were soaked in a cryoprotectant solution consisting of crystallization mother liquor with increasing concentrations of PEG 6000, sucrose, and glycerol up to 10%, 5%, and 25%, respectively. For preparation of the RT^Q151M^:DNA:dGTP and RT^Q151M^:DNA:ETV-TP ternary complex, the crystals were further soaked briefly in the same cryoprotectant solution supplemented with 2.4 mM dGTP/ETV-TP. The crystals were flash-cooled and stored in liquid nitrogen for the X-ray diffraction experiments.

### Structure determination and refinement

X-ray diffraction data sets were collected using synchrotron radiation at BL-17A and BL-1A beamlines of the Photon Factory (Tsukuba, Japan). X-ray diffraction data were processed using the program XDS^40^. The crystals belonged to rhombohedral space group *R*3, with unit-cell parameters a = b = 284, c = 96 Å. The structure was solved by molecular replacement using the program MOLREP^41^, with the previously reported HIV-1 RT^WT^ structure in complex with the DNA aptamer as a search model (PDB code, 5D3G)^27^. The atomic model was fitted manually, using the program Coot^42^. Model refinement was performed using the programs REFMAC5^43^ and Phenix^44^. An atomic model of ETV-TP was created using the program SKETCHER, and the geometrical restraints files were generated using the LIBCHECK module provided in the CCP4 program package^45^. Data collection and refinement statistics are summarized in Supplementary Table S3. MolProbity software^46^ was used for model validation.

### Data availability statements

The atomic coordinates and structure factor amplitudes of the RT^Q151M^:DNA, RT^Q151M^:DNA:ETV-TP, and RT^Q151M^:DNA:dGTP complexes have been deposited in the RCSB Protein Data Bank (http://www.rcsb.org) under accession codes 5XN0, 5XN1, and 5XN2, respectively. Other data generated and/or analyzed during this study are available from the corresponding authors upon reasonable request.

## Electronic supplementary material

Supplementary Information

## References

[1] Lavanchy D (2004). Hepatitis B virus epidemiology, disease burden, treatment, and current and emerging prevention and control measures. J. Viral. Hepat..

[2] Wang JC-Y, Nickens DG, Lentz TB, Loeb DD, Zlotnick A (2014). Encapsidated hepatitis B virus reverse transcriptase is poised on an ordered RNA lattice. Proc. Natl. Acad. Sci. USA.

[3] Seeger C, Mason WS (2000). Hepatitis B virus biology. Microbiol. Mol. Biol. Rev..

[4] Summers J, Mason WS (1982). Replication of the genome of a hepatitis B-like virus by reverse transcription of an RNA intermediate. Cell.

[5] Zoulim F, Seeger C (1994). Reverse transcription in hepatitis B viruses is primed by a tyrosine residue of the polymerase. J. Virol..

[6] Weber M (1994). Hepadnavirus P protein utilizes a tyrosine residue in the TP domain to prime reverse transcription. J. Virol..

[7] Vörös J (2013). Large-scale production and structural and biophysical characterizations of the human hepatitis B virus polymerase. J. Virol..

[8] Nassal M (2009). New insights into HBV replication: new opportunities for improved therapies. Future Virol..

[9] Shirasaka T (1993). Changes in drug sensitivity of human immunodeficiency virus type 1 during therapy with azidothymidine, dideoxycytidine, and dideoxyinosine: an *in vitro* comparative study. Proc. Natl. Acad. Sci. USA.

[10] Shirasaka T (1995). Emergence of human immunodeficiency virus type-1 variants with resistance to multiple dideoxynucleosides in patients receiving therapy with dideoxynucleosides. Proc. Natl. Acad. Sci. USA.

[11] Das K, Martinez SE, Arnold E (2017). Structural insights into HIV reverse transcriptase mutations Q151M and Q151M complex that confer multi-nucleoside drug resistance. Antimicrob. Agents Chemother..

[12] Woo G (2010). Tenofovir and entecavir are the most effective antiviral agents for chronic hepatitis B: a systematic review and Bayesian meta-analyses. Gastroenterology.

[13] Michailidis E (2012). Antiviral therapies: focus on hepatitis B reverse transcriptase. Int. J. Biochem. Cell Biol..

[14] Domaoal RA (2008). Pre-steady-state kinetic studies establish entecavir 5’-triphosphate as a substrate for HIV-1 reverse transcriptase. J. Biol. Chem..

[15] Seifer M, Hamatake RK, Colonno RJ, Standring DN (1998). *In vitro* inhibition of hepadnavirus polymerases by the triphosphates of BMS-200475 and lobucavir. Antimicrob. Agents Chemother..

[16] Tchesnokov EP, Obikhod A, Schinazi RF, Gotte M (2008). Delayed chain termination protects the anti-hepatitis B virus drug entecavir from excision by HIV-1 reverse transcriptase. J. Biol. Chem..

[17] Hayashi S (2015). Characterization of novel entecavir resistance mutations. J. Hepatol..

[18] Mukaide M (2010). Mechanism of entecavir resistance of hepatitis B virus with viral breakthrough as determined by long-term clinical assessment and molecular docking simulation. Antimicrob. Agents Chemother..

[19] Langley DR (2007). Inhibition of hepatitis B virus polymerase by entecavir. J. Virol..

[20] Innaimo SF (1997). Identification of BMS-200475 as a potent and selective inhibitor of hepatitis B virus. Antimicrob. Agents Chemother..

[21] Lin PF (2008). Entecavir exhibits inhibitory activity against human immunodeficiency virus under conditions of reduces viral challenge. Antimicrob. Agents Chemother..

[22] Harada S, Hazra R, Tamiya S, Zeichner SL, Mitsuya H (2007). Emergence of human immunodeficiency virus type 1 variants containing the Q151M complex in children receiving long-term antiretroviral chemotherapy. Antiviral Res..

[23] Boyer PL, Sarafianos SG, Clark PK, Arnold E, Hughes SH (2006). Why do HIV-1 and HIV-2 use different pathways to develop AZT resistance?. PLoS Pathog..

[24] B PL. Clark PK, Hughes SH (2012). HIV-1 and HIV-2 reverse transcriptases: different mechanisms of resistance to nucleoside reverse transcriptase inhibitors. J. Virol..

[25] Margot NA, Johnson A, Miller MD, Callebaut C (2015). Characterization of HIV-1 resistance to tenofovir alafenamide *in vitro*. Antimicrob. Agents Chemother..

[26] DeStefano JJ, Cristofaro JV (2006). Selection of primer-template sequences that bind human immunodeficiency virus reverse transcriptase with high affinity. Nucleic Acids Res..

[27] Miller MT, Tuske S, Das K, DeStefano JJ, Arnold E (2015). Structure of HIV-1 reverse transcriptase bound to a novel 38-mer hairpin template-primer DNA aptamer. Protein Sci..

[28] Salie ZL (2016). Structural basis of HIV inhibition by translocation-defective RT inhibitor 4’-ethynyl-2-fluoro-2’-deoxyadenosine (EFdA). Proc. Natl. Acad. Sci. USA.

[29] Das K, Martinez SE, Bauman JD, Arnold E (2012). HIV-1 reverse transcriptase complex with DNA and nevirapine reveals non-nucleoside inhibition mechanism. Nat. Struct. Mol. Biol..

[30] McMahon MA (2007). The HBV drug entecavir-effects on HIV-1 replication and resistance. N. Engl. J. Med..

[31] Maeda Y, Venzon DJ, Mitsuya H (1998). Altered drug sensitivity, fitness, and evolution of human immunodeficiency virus type 1 with pol gene mutations conferring multi-dideoxynucleoside resistance. J. Infect. Dis..

[32] Takamatsu Y (2015). 4′-modified nucleoside analogs: potent inhibitors active against entecavir-resistant hepatitis B virus. Hepatology.

[33] Hemsley A, Arnheim N, Toney MD, Cortopassi G, Galas DJ (1989). A simple method for site-directed mutagenesis using the polymerase chain reaction. Nucleic Acids Res..

[34] Nakamura A, Tamura N, Yasutake Y (2015). Structure of the HIV-1 reverse transcriptase Q151M mutant: insights into the inhibitor resistance of HIV-1 reverse transcriptase and the structure of the nucleotide-binding pocket of Hepatitis B virus polymerase. Acta Crystallogr. Sect. F.

[35] Maeda K (2014). Delayed emergence of HIV-1 variants resistant to 4′-ethynyl-2-fluoro-2′-deoxyadenosine: comparative sequential passage study with lamivudine, tenofovir, emtricitabine and BMS-986001. Antivir. Ther..

[36] Maeda K (2001). Novel low molecular weight spirodiketopiperazine derivatives potently inhibit R5 HIV-1 infection through their antagonistic effects on CCR5. J. Biol. Chem..

[37] Maeda M, Patel AD, Hampton A (1977). Formation of ribonucleotide 2′,3′-cyclic carbonates during conversion of ribonucleoside 5′-phosphates to diphosphates and triphosphates by the phosphorimidazolidate procedure. Nucleic Acids Res..

[38] Bauman JD (2008). Crystal engineering of HIV-1 reverse transcriptase for structure-based drug design. Nucleic Acids Res..

[39] Vermeire J (2012). Quantification of reverse transcriptase activity by real-time PCR as a fast and accurate method for titration of HIV, lenti- and retroviral vectors. PLoS One.

[40] Kabsch WX (2010). Acta Crystallogr. D Biol. Crystallogr..

[41] Vagin A, Teplyakov A (2010). Molecular replacement with MOLREP. Acta Crystallogr. Sect. D.

[42] Emsley P, Lohkamp B, Scott WG, Cowtan K (2010). Features and development of Coot. Acta Crystallogr. Sect. D.

[43] Murshudov GN (2011). REFMAC5 for the refinement of macromolecular crystal structures. Acta Crystallogr. Sect. D.

[44] Adams PD (2010). Phenix: a comprehensive Python-based system for macromolecular structure solution. Acta Crystallogr. Sect. D.

[45] Winn MD (2011). Overview of the CCP4 suite and current developments. Acta Crystallogr. Sect. D.

[46] Chen VB (2010). MolProbity: all-atom structure validation for macromolecular crystallography. Acta Crystallogr. Sect. D.

