# Supplementary material for: HIV-1 with HBV-associated Q151M substitution in RT becomes highly susceptible to entecavir: structural insights into HBV-RT inhibition by entecavir
Source: Sci Rep. 2018 Jan 26;8:1624. doi: 10.1038/s41598-018-19602-9 (PMC5785976; doi:10.1038/s41598-018-19602-9)
Supplement: Supplementary file 1 — Supplementary Information [file 41598_2018_19602_MOESM1_ESM.pdf]

Supplementary information

HIV-1 with HBV-associated Q151M substitution in RT becomes highly susceptible to entecavir: structural insights into HBV-RT inhibition by entecavir

Yoshiaki Yasutake[1]*, Shin-ichiro Hattori[2], Hironori Hayashi[2], Kouki Matsuda[2], Noriko Tamura[1], Satoru Kohgo[2,3], Kenji Maeda[2]*, Hiroaki Mitsuya[2,4,5]

[1]Bioproduction Research Institute, National Institute of Advanced Industrial Science and Technology (AIST), Sapporo 062-8517, Japan.
[2]National Center for Global Health and Medicine Research Institute, Tokyo 162-8655, Japan.
[3]Faculty of Pharmaceutical Sciences, Sojo University, Kumamoto 860-0082, Japan.
[4]Experimental Retrovirology Section, HIV and AIDS Malignancy Branch, National Cancer Institute, National Institutes of Health, Bethesda, MD 20892, USA.
[5]Department of Infectious Diseases, Graduate School of Medical Sciences, Kumamoto University, Kumamoto 860-8556, Japan.

*Correspondence should be addressed to Yoshiaki Yasutake. Tel: +81-11-857-8514; Fax: +81-11-857-8980; E-mail: y-yasutake@aist.go.jp. Correspondence may also be addressed to Kenji Maeda. Tel: +81-3-3202-7181; Fax: +81-3-3207-1038; E-mail: kmaeda@ri.ncgm.go.jp.

Supplementary Table S1. Results of the enzyme assay for HIV-1 RT^{WT} and RT^{Q151M}

	RT activity (mU/ng)	
	ELISA	PCR
RT^{WT}	6.23	2.58
RT^{Q151M}	6.16 (0.99)	1.99 (0.77)

The parentheses refer to the values of relative enzymatic activity of HIV-1 RT^{Q151M} compared to that of RT^{WT}.

Supplementary Table S2. Inter-atomic distances representing the relative ETV-TP binding position compared to those of other typical dNTPs and NRTIs in HIV-1 RT

PDB	NRTI/ dNTP	Chain	Distance *X* (Å)	Distance *Y* (Å)	Resolution (Å)	Ref.
5XN1	ETV-TP	A	4.07	6.00	2.45	This study.
		C	4.16	5.82		
5XN2	dGTP	A	3.77	4.42	2.38	This study.
		C	3.80	4.63		
5TXL	dATP	A	3.54	4.14	2.50	(11)
		C	3.37	4.07		
5TXN	dATP	A	3.56	4.21	2.55	(11)
		C	3.32	4.05		
5TXO	dATP	A	3.44	4.31	2.70	(11)
		C	3.31	4.26		
5J2M	EFdA-TP	A	3.32	4.90	2.43	(28)
3V4I	AZT-TP	A	3.35	4.24	2.80	(29)
		C	3.53	4.16		

Distance *X* represents the interatomic distances (Å) between C6′/O4′ atoms of dNTP/NRTI at the N-site and the C2′ atoms of the 3′-end nucleotide. Distance *Y* represents the interatomic distances (Å) between C6′/O4′ of dNTP/NRTI at the N-site and the Met184 SD atoms.

Supplementary Table S3. Crystallographic parameters and refinement statistics

	RT^{Q151M}:DNA	RT^{Q151M}:DNA: dGTP	RT^{Q151M}:DNA: ETV-TP
**PDB code**	5XN0	5XN2	5XN1
**Data collection**			
Beamline	PF BL-17A	PF BL-1A	PF BL-17A
Detector	Pilatus3S 6M	Eiger X4M	Pilatus3S 6M
Wavelength (Å)	0.98000	1.10000	0.98000
Space group	*R*3	*R*3	*R*3
Unit-cell parameters (Å)	$a = b = 284.09$, $c = 95.61$	$a = b = 285.69$, $c = 96.33$	$a = b = 284.71$, $c = 95.79$
Resolution (Å)*	50-2.60 (2.75–2.60)	50-2.38 (2.52–2.38)	50-2.45 (2.59–2.45)
Unique reflections	88,842	117,205	106,838
R_{meas}*,†	0.084 (0.758)	0.063 (0.818)	0.113 (0.834)
Mean $I/\sigma(I)$*	16.9 (2.5)	20.6 (2.0)	12.0 (2.1)
Completeness (%)*	100.0 (99.8)	99.8 (99.0)	99.9 (99.4)
Multiplicity*	5.2 (5.0)	5.3 (5.3)	5.3 (5.0)
**Refinement**			
R_{work}/R_{free}‡,§	0.185/0.226	0.189/0.223	0.188/0.227
No. of atoms	17,318	17,606	17,562
Mean *B*-factors ($Å^2$)			
All/DNA	71.6/68.7	69.0/66.9	59.0/55.5
dNTP/NRTI	-	72.2 (dGTP)	65.8 (ETV-TP)
R.m.s.d. from ideal			
Bond lengths (Å)	0.009	0.003	0.009
Bond angles (°)	1.14	0.595	0.90
Ramachandran plot¶			
Favored	97.11	97.01	96.27
Allowed	2.78	2.78	3.57
Outliers (%)	0.11	0.21	0.16

* Values in parentheses are for the outermost resolution shell.

† $R_{meas} = \{\Sigma_h [N_h / (N_h - 1)]^{1/2} \Sigma_i |I_{h,i} - \langle I_h \rangle|\} / \Sigma_h \Sigma_i I_{h,i}$, where $\langle I_h \rangle$ is the mean intensity of the observations $I_{h,i}$ of reflection h, and N_h is the multiplicity of reflection h.

‡ $R_{work} = \Sigma |F_{obs} - F_{calc}| / \Sigma F_{obs}$ for 95% of the reflection data used in the refinement. F_{obs} and F_{calc} are the observed and calculated structure factor amplitudes, respectively.

§ R_{free} is the equivalent of R_{work}, except that it was calculated for a randomly chosen 5% test set excluded from refinement.

¶ Ramachandran analysis was performed using the program MolProbity[46].

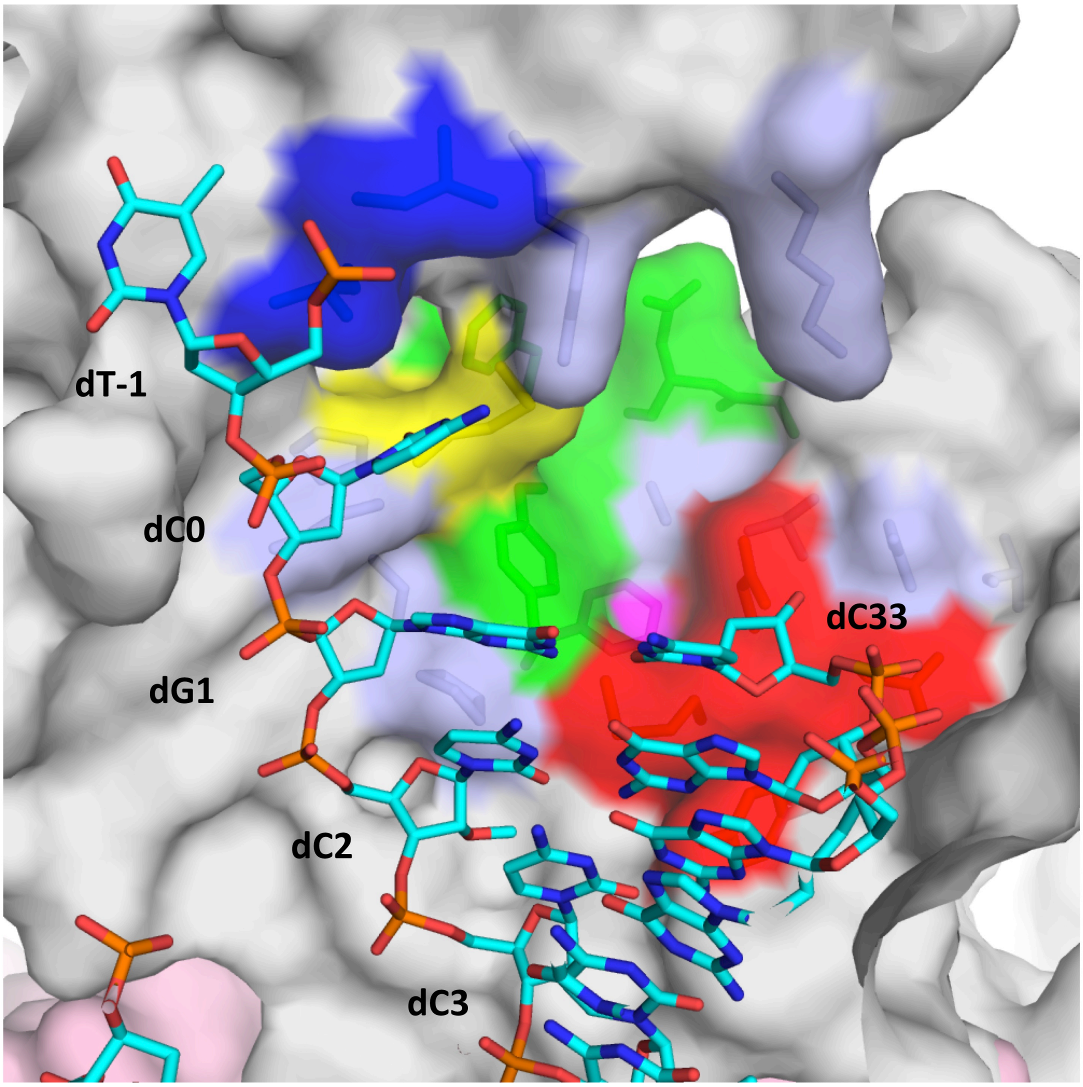

Supplementary Fig. S1. Molecular surface representation of the N-site indicating the location of mutational residues. The structure of the HIV-1 RT^{Q151M}:DNA binary complex was used for the calculation of molecular surface. The mutational residues are colored with the same color scheme used in Fig. 1. The conserved YMDD residues in the motif C are colored in red. Additional conserved residues between HIV-1 and HBV RT nearby the N-site (Lys65, Arg72, Leu109, Asp110, Val111, Ala114, Pro150, Gly152, Ser156, and Pro157) are also shown in light blue.

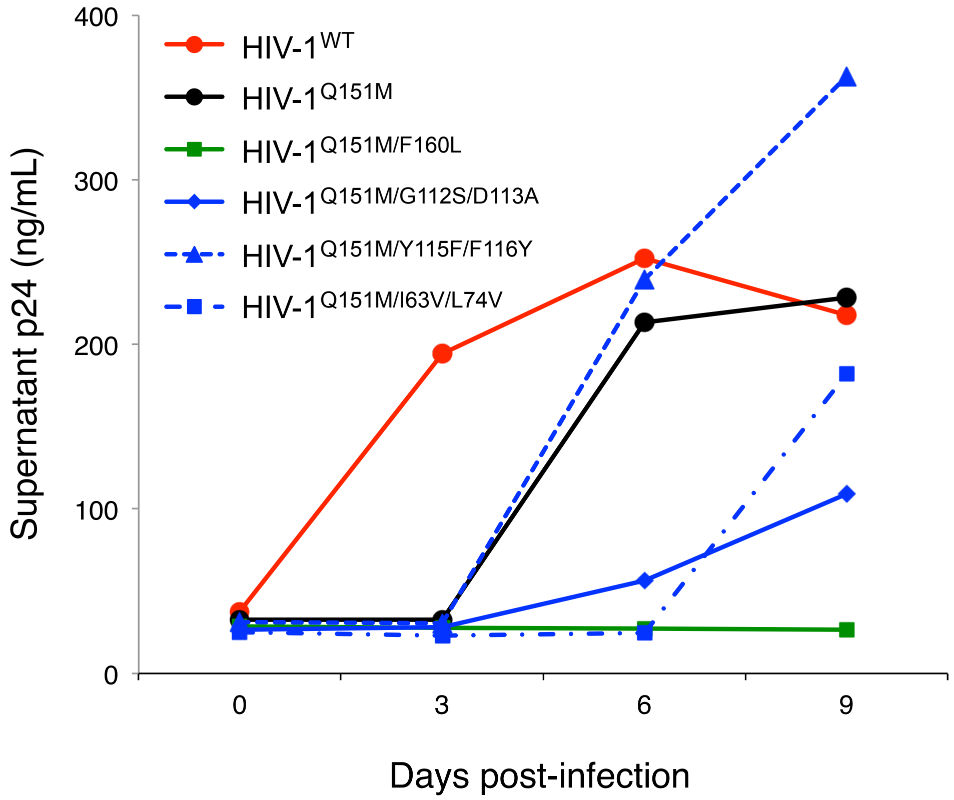

Supplementary Fig. S2. Replication kinetics of HIV-1^{WT} and the HIV-1 variants (HIV-1^{Q151M}, HIV-$1^{Q151M/F160L}$, HIV-$1^{Q151M/G112S/D113A}$, HIV-$1^{Q151M/Y115F/F116Y}$, and HIV-$1^{Q151M/I63V/L74V}$). The p24 values in supernatants were determined on days 0, 3, 6, and 9.

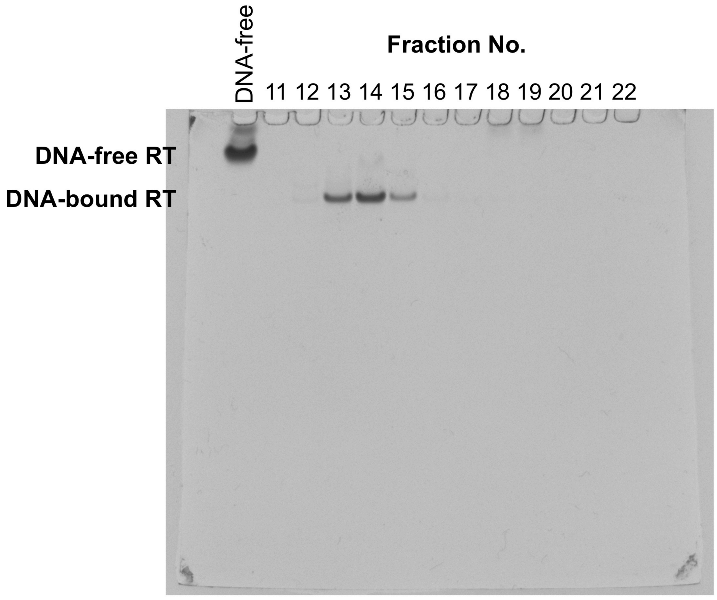

CBB staining (protein)

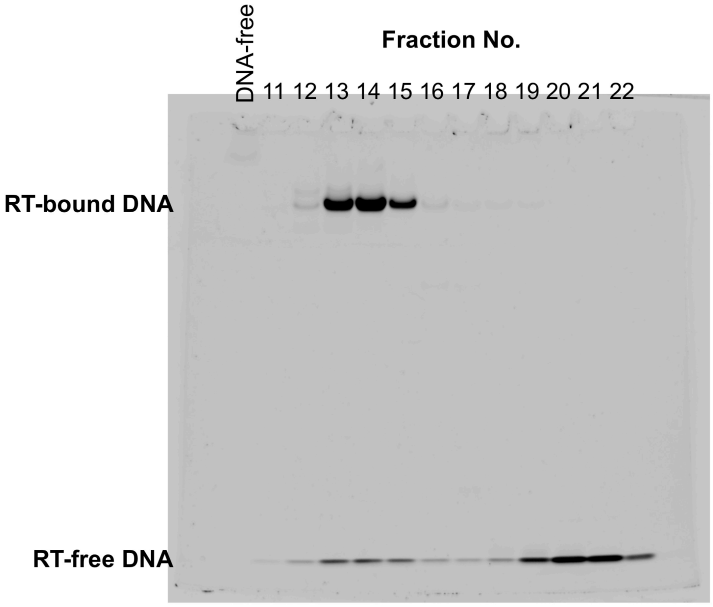

Fluorescent staining (DNA)

Supplementary Fig. S3. Native PAGE analysis of the fractions of gel-filtration chromatography. DNA-free, the RT^{Q151M} sample before DNA complex formation (DNA-free RT). Fractions #11–22, the fractions of gel-filtration chromatography including the top elution peak. The same gel was used for both protein and DNA detection (see Methods). Fractions #13 and #14 were collected for crystallization.

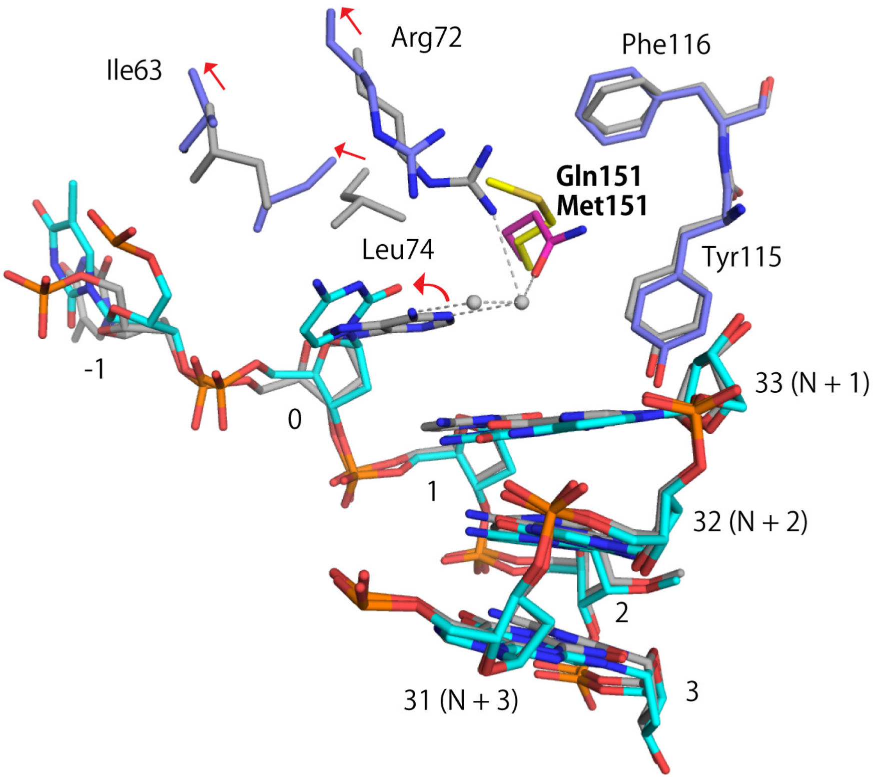

Supplementary Fig. S4. Structure superimposition of two HIV-1 RT:DNA aptamer binary complexes. The previously reported HIV-1 RT^{WT}:DNA (PDB code, 5D3G)[27] is shown in gray for carbon atoms, whereas the side-chain of Gln151 is colored in magenta. Two solvent molecules that stabilize the base moiety of the template strand at position 0 are also shown. Hydrogen bonds are drawn as dotted lines. The present HIV-1 RT^{Q151M}:DNA complex is shown in light blue for protein carbons and in cyan for DNA carbon atoms. Met151 is colored in yellow. Bulky and hydrophobic Met151 side-chain keeps nearby residues away, leading to the more open conformation. The apparent structural changes are indicated as red arrows.

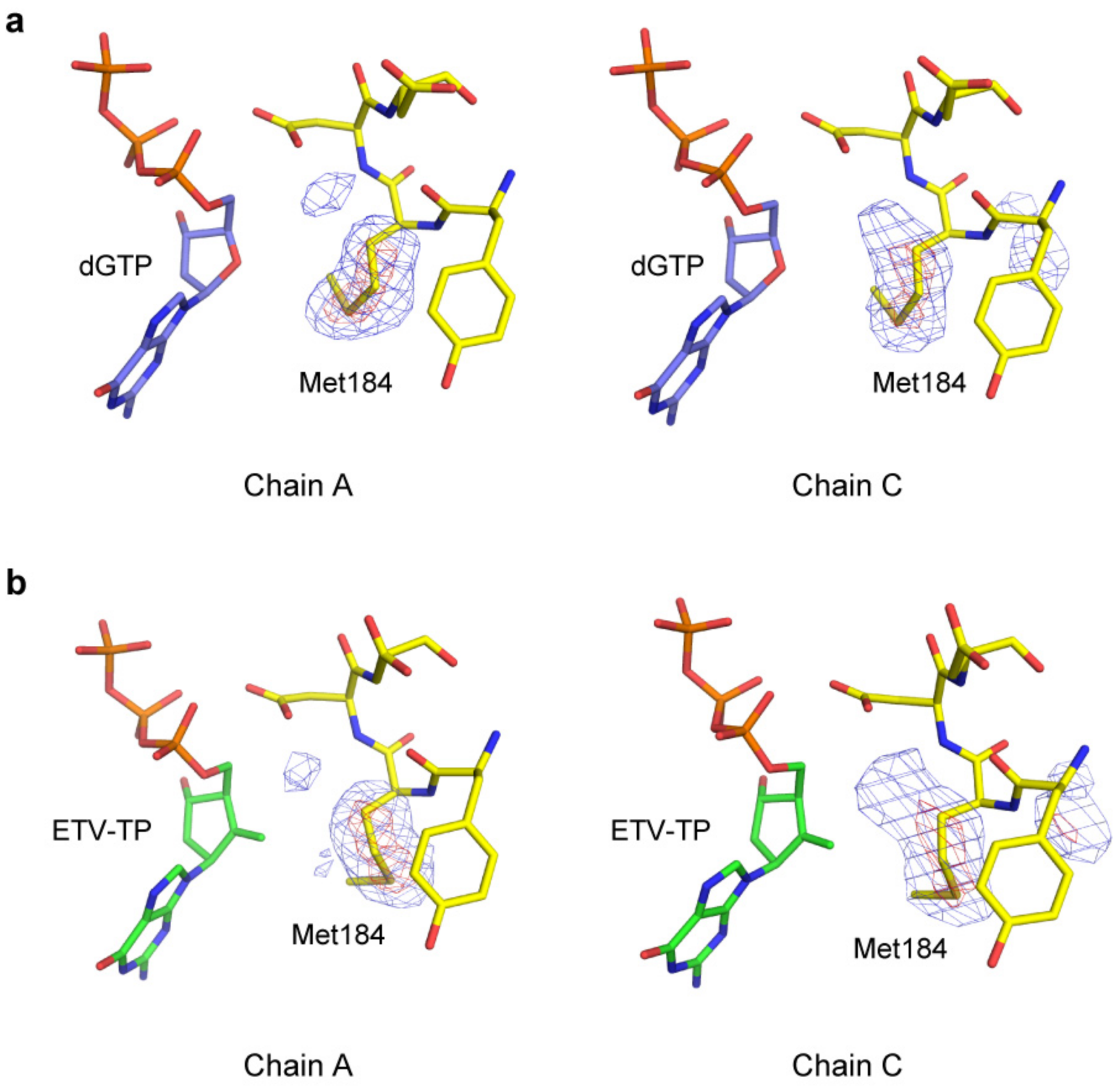

Supplementary Fig. S5. Simulated annealing $F_o - F_c$ omit map for the Met184 side chains of (a) RT^{Q151M}:DNA:dGTP and (b) RT^{Q151M}:DNA:ETV-TP ternary complex, contoured at 2.6σ in blue and at 5.0σ level in red. Both chains A and C of each ternary complex structure are presented. The model of the YMDD motif in motif C (Fig. 1c) including Met184 is shown as a stick model.

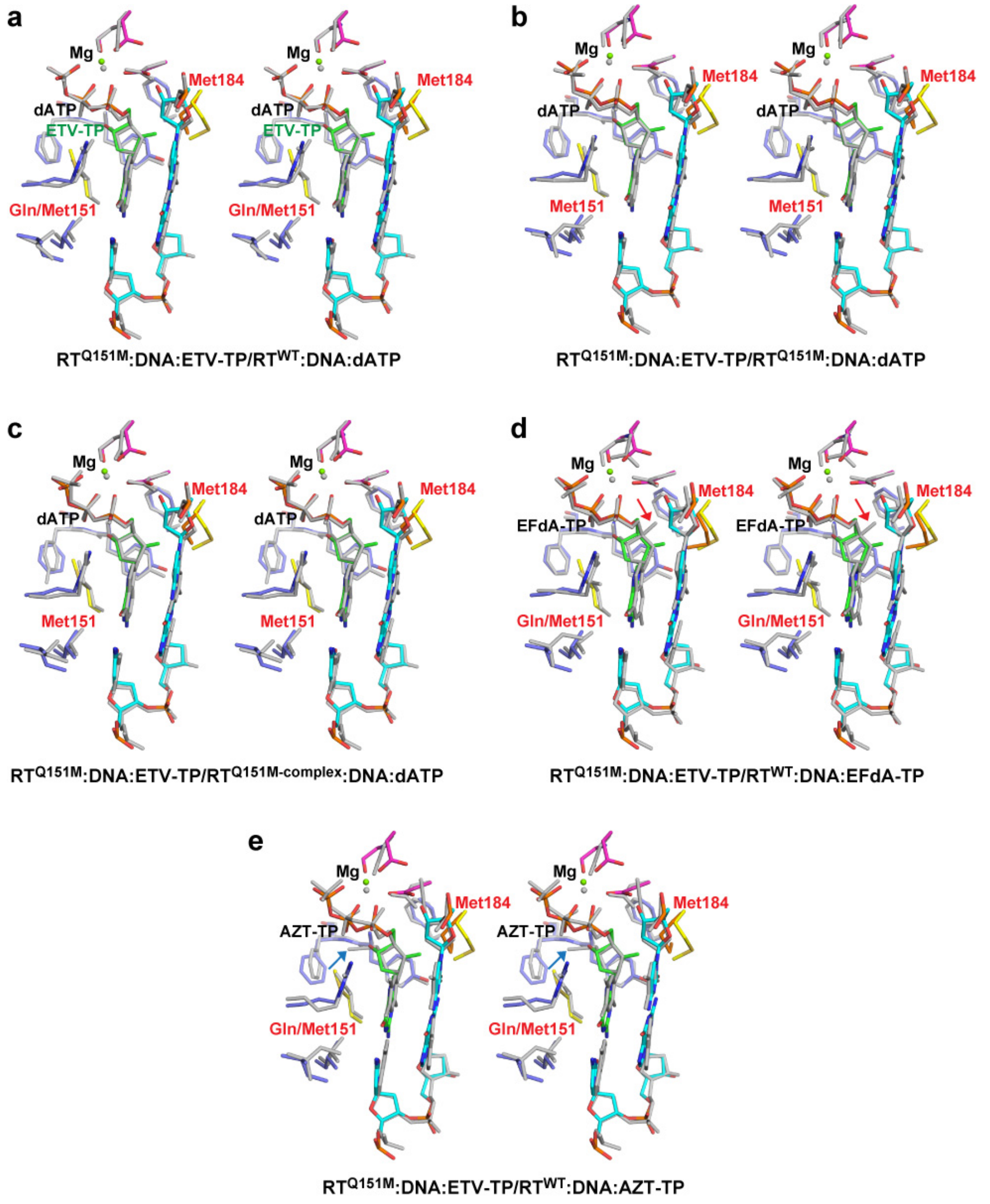

Supplementary Fig. S6. Stereo-view structure superimposition of the N-site between RT^{Q151M}:DNA:ETV-TP and the previously reported ternary complex of (a) RT^{WT}:DNA:dATP (PDB code, 5TXL), (b) RT^{Q151M}:DNA:dATP (PDB code, 5TXN), (c) $RT^{Q151M\text{-complex}}$:DNA:dATP (PDB code, 5TXO)[11], (d) RT^{WT}:DNA:EFdA-TP (PDB code, 5J2M)[28] and (e) RT^{WT}:DNA:AZT-TP (PDB code, 3V4I)[29]. The structure of RT^{Q151M}:DNA:ETV-TP is colored according to the same scheme as Fig. 5. The other superimposed structures are colored in gray. Key residues of Met184 and Gln/Met151 are indicated in red. The 4′-ethynyl group is indicated by a red arrow. Also, the 3′-azide group is indicated by a blue arrow.

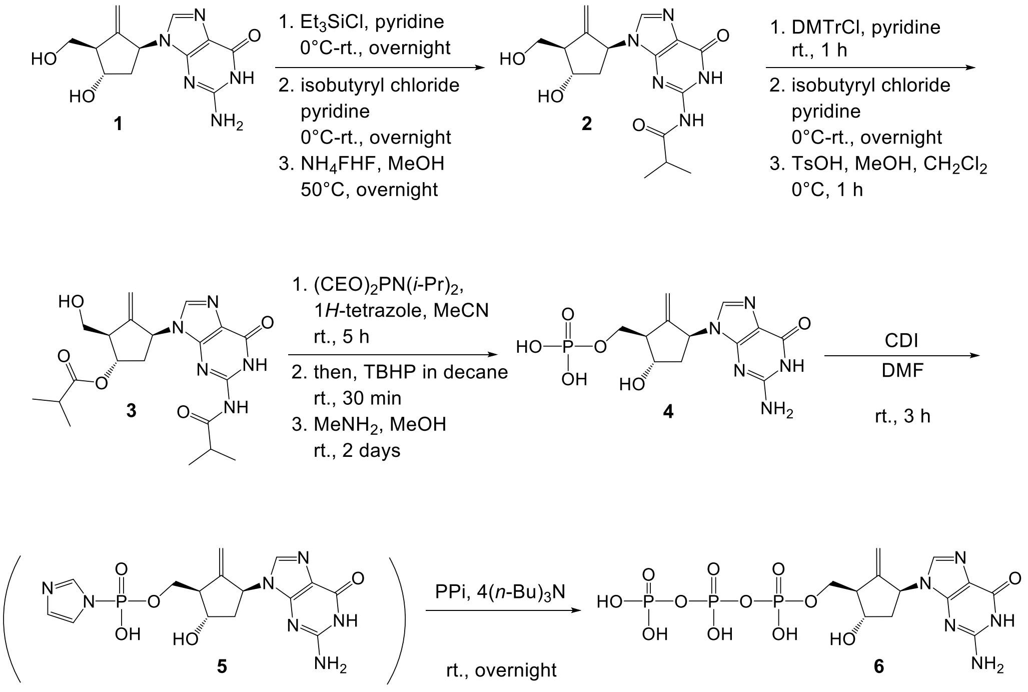

Supplementary Fig. S7. Scheme for the synthesis of ETV-TP.
